# Empowerment and use of modern contraceptive methods among married women in Burkina Faso: a multilevel analysis

**DOI:** 10.1186/s12889-021-11541-x

**Published:** 2021-08-03

**Authors:** Sylvain Y. M. Some, Christy Pu, Song-Lih Huang

**Affiliations:** 1 International Health Program, National Yang Ming Chiao Tung University, Taipei, Taiwan, Republic of China; 2 International Health Program, Institute of Public Health, School of Medicine, National Yang Ming Chiao Tung University, No. 155, Sec. 2, Linong St. 11221, Taipei City, Taiwan, Republic of China

**Keywords:** Gender inequality, women’s empowerment, Family planning, Burkina Faso

## Abstract

**Background:**

In Burkina Faso, gender inequality prevents women from meeting their reproductive needs, leading to high rates of unintended pregnancies, abortions and deaths. Evidence shows that empowering women may increase the proportion of demand for family planning satisfied using modern methods (mDFPS), but few studies have measured this process in multiple spheres of life. We investigated how empowerment influences the mDFPS among married women of reproductive age (MWRA) in Burkina Faso.

**Methods:**

We analyzed data from the 2010 Burkina Faso Demographic and Health Survey (DHS) on 4714 MWRA with reproductive needs living in 573 communities. We used principal component analysis (PCA) and Cronbach’s alpha test to explore and assess specific and consistently relevant components of women’s agency in marital relationships. Aggregated measures at the cluster level were used to assess gender norms and relationships in communities. Descriptive statistics were performed and multilevel logistic regression models were carried out to concurrently gauge the effects of women’s agency and community-level of gender equality on mDFPS, controlling for socioeconomic factors.

**Results:**

Overall, less than one-third (30.8%) of the demand for family planning among MWRA were satisfied with modern methods. Participation in household decision-making, freedom in accessing healthcare, and opposition to domestic violence were underlying components of women’s agency in marital relationships. In the full model adjusted for socioeconomic status, freedom in accessing healthcare was significantly (aOR 1.27, CI 1.06–1.51) associated with mDFPS. For community-level variables, women’s greater access to assets (aOR 1.72, 95% CI 1.13–2.61) and family planning messages (aOR 2.68, 95% CI 1.64–4.36) increased mDFPS, while higher fertility expectations (aOR 0.75, 95% CI 0.64–0.87) reduced it. Unexpectedly, women in communities with higher rates of female genital mutilation were more likely (aOR 2.46, 95% CI 1.52–3.99) to have mDFPS.

**Conclusions:**

Empowering women has the potential to reduce gender inequality, raise women’s agency and increase mDFPS. This influence may occur through both balanced marital relationships and fair community gender norms and relationships. Progress toward universal access to reproductive services should integrate the promotion of women’s rights.

**Trial registration:**

No clinical trial has been performed in this study.

**Supplementary Information:**

The online version contains supplementary material available at 10.1186/s12889-021-11541-x.

## Background

Achieving universal access to sexual and reproductive health and rights is a key component to achieving Sustainable Development Goals (SDGs) 3 and 5, which aim to ensure healthy lives and wellbeing for all as well as gender equality [1]. In sub-Saharan Africa, the modern contraceptive prevalence rate doubled among all married women of reproductive age (MWRA), from 14.7% in 2000 to 27.9% in 2019 [2]. However, only 52% of those in need of family planning used modern methods. Consequently, 14 million unintended pregnancies are recorded each year, leading to unsafe abortions, maternal deaths and socioeconomic loss [3, 4].

Despite investment in family planning programs and an increase in education attainment, African women are disproportionally deprived of the means to meet their family planning needs [5]. Economic, cultural and geographical disparities that hinder the promotion of family planning also prevent modern contraceptive decisions [6, 7]. Evidence shows that increasing women’s access to resources and rights has the potential to empower them to make decisions to use modern contraceptives and reduce fertility [8]. Contraceptive decision-making is primarily made in marital relationships, but it is also influenced by community norms about gender roles and relationships [9, 10].

In 2015, the SDGs introduced an indicator of family planning performance, i.e., the percent of demand for family planning satisfied with modern methods (mDFPS), with the goal of achieving at least 75% mDFPS by 2030 [11]. A major difference between the proportion of mDFPS and the previously measured modern contraceptive prevalence rate is that this new measure only targets women who need family planning, therefore explicitly recognizes women’s right to control their own fertility and their autonomy to decide on effective modern methods [12]. To date, among low- and middle-income countries (52.9%), West and Central Africa (32.9%) lag behind other areas in terms of mDFPS [13]. In particular, Burkina Faso, a West African country, has seen the highest increase in the proportion of MWRA unable to meet their reproductive needs, from 26.5 in 1990 to 30.2 in 2010 [14]. As a result, the proportion of mDFPS was only 40% in 2010, and projections estimate that it will reach 52% by 2030, which is only two-thirds of the proposed target in the SDGs [15].

Until recently, research in Africa has focused on women’s socioeconomic status and family planning services as major determinants of contraceptive use [16, 17]. For instance, in Burkina Faso, women’s modern contraceptive use was found to be positively associated with wealth, educational attainment, asset ownership, smaller ideal family size, monogamy, the presence of a living son, and the presence of a child younger than 1 year [18–20]. In addition, living in a more urbanized location, living near a health facility and having been visited by a community health worker were significant factors related to a higher odd of modern contraception [21–24]. Nevertheless, these studies did not adequately consider both women’s ability to make reproductive decisions and the community gender norms that affect these decisions [24–26]. Recently, the literature has reported that a greater division in gender roles and relationships affects women’s autonomy and decreases their ability to obtain effective contraception [9, 10]. Metheny and Stephenson [27] found that women who lived in communities with greater men’s educational attainment, lower female employment, higher justification of domestic violence, a higher ideal number of children, and lower wealth were less likely to use contraceptives. Furthermore, SDG 5 outlined structural targets to achieve gender equality and empower all women and girls, including ending all forms of violence and discrimination against women and ensuring their access to rights and opportunities [1]. Despite this evidence, few studies have simultaneously addressed the influence of gender on communities and individuals as well as modern contraceptive use in the African context [25, 28]. Additionally, there is a lack of knowledge on systematic measures of imbalanced marital relationships and community-level gender inequality that may affect the process of empowerment for modern contraceptive use among women who need family planning.

Our theoretical framework drew on socioecological theory, which recognizes the key influence of factors at the individual, household and community levels on health behaviors [29, 30]. In the context of gender inequality, the traditional division of gender roles and relationships based on sex limits women’s ability to access resources and use modern contraceptives, even though they need family planning [28]. These limits may translate into violence against women, deprivation of the rights and opportunities enjoyed by women in communities, and an imbalance in marital relationships. In fact, the process of women’s empowerment is about gaining the ability to make free choices, autonomous decisions and achieve desired outcomes despite deep-seated limits [31]. Women’s empowerment is commonly conceptualized as resources, agency and achievements [32]. Resources are material and non-material assets to the enhancement of agency then the transformation of choices into achievements. Agency, as a central part of the process, refers to women’s ability to define one’s goals and act upon them. For instance, improving women’s access to resources through gender equality would not lead to empowerment unless women act as agents of change rather than mere recipients. Nevertheless, the exercise of agency become meaningful when it contributes to achieve women’s well-being either through a social struggle or a shift in gender relationships [33, 34].

Measuring women’s empowerment remains challenging due to the latent and context-specific nature of agency. Using factor analysis, an explorative study on women’s agency identified multiple domains as follows participation in family decisions, freedom of movement, and vocalization of more gender equitable attitudes [33]. However, mixed results have been found regarding these domains in association with modern contraceptive use, which has led to their relevance and consistency, especially in the African context, to be questioned [35, 36]. In fact, the pronatalist nature of African societies prescribes strict gender roles and relationships that deprive women’s access to resources, rights, and opportunities to productive activities and confine them to reproductive roles [6].

We study this issue in Burkina Faso, where not only are gender inequality indices among the worst in West Africa but MWRA are still unable to meet their needs for family planning [14, 37].

The aim of this study is to first explore and identify relevant and consistent components of women’s agency in marital relationships, then assess community-level gender norms and relationships, and finally, examine their association with modern contraceptive use among MWRA in Burkina Faso. This approach allows a better description of how gender equality may influence women’s ability to make decisions about modern contraceptive use. This knowledge may help in designing comprehensive interventions to accelerate universal access to modern contraceptives, thereby aiding progress toward the SDGs.

## Methods

### Source of data

Data were retrieved from individual women’s files available from the 2010 Burkina Faso Demographic and Health Survey (DHS) [38]. This survey collected nationally representative data on women’s empowerment, modern contraceptive use and socioeconomic characteristics. It employed a two-stage cluster design stratified by urban and rural areas. The first stage consisted of sampling clusters based on the enumeration areas (EAs) delineated in the 2006 population and household census. A total of 574 clusters were selected by probability proportional to size sampling; 176 of the clusters were in urban areas, and 378 were in rural areas. However, one EA located in the Sahel region was not surveyed. The second stage involved the systematic sampling of approximately 25 households from each cluster, which yielded a total sample of 14,947 households; among these households, 14,424 were interviewed. All women aged 15–49 years in the households were eligible, yielding a final sample of 17,087 women from 14,242 households and 573 clusters, with response rates of 99.8, 99.2 and 98.4%, respectively.

### Population

**Fig. 1:**
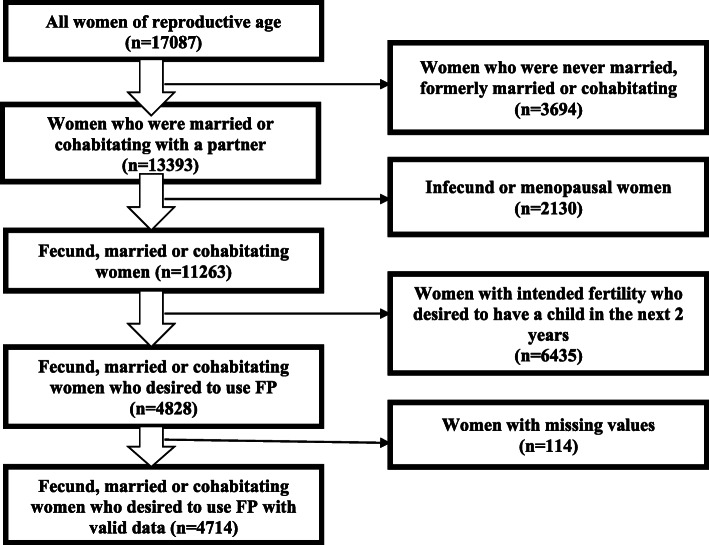
Flow chart of the sample selection

The use of contraceptive methods was measured among MWRA aged 15–49 years with family planning needs, that is, those who wanted to avoid unintended pregnancies. According to the DHS, MWRA are women who are fecund, are currently married, and want to postpone their next birth for two or more years or to stop childbearing altogether. However, the definition also includes pregnant women whose pregnancies are mistimed or unwanted and amenorrhoeic women whose last birth was mistimed or whose last child was unwanted. After the removal of missing values, the unweighted final population included 4714 MWRA at risk of or with experience of unintended pregnancies (Fig. 1).

### Outcome variable

The outcome variable was mDFPS among MWRA. To assess the use of contraceptive methods, the DHS first asked about any current use of contraception and then the type of method used. According to the DHS, modern contraceptive methods include female and male sterilization, intrauterine devices, injectables, implants, the pill, male and female condoms, the lactational amenorrhea method, emergency contraception, diaphragms, foams and jellies [38]. Rhythm and withdrawal are considered traditional methods. The outcome variable is binary. It takes the value “0” if a woman used a traditional method or did not use contraception and “1” if a woman used a modern method.

### Exploratory variable

Our main exploratory variable is empowerment, measured at the community level as factors of gender-based discrimination and opportunity and measured in marital relationships as women’s agency.

Based on previous research, we explored the following relevant and context-specific components of women’s agency: influence in family decisions, attitudes toward domestic violence and freedom of movement in public spaces [33]. In the 2010 DHS, the ability to participate in household decision-making was an essential indicator of agency. It was assessed based on women’s autonomy (jointly or alone) to decide on their own health care, make major household purchases, and visit family or relatives. However, this autonomy could be overridden when they faced domestic violence. Another section of the survey instrument assessed women’s attitudes toward domestic violence by asking whether (yes, no, or don’t know) a husband is justified in hitting or beating his wife in each of the following five situations: if she burns food, if she argues with him, if she goes out without telling him, if she neglects the children, and if she refuses to have sexual intercourse with him. Furthermore, other factors may prevent women from accessing healthcare during their reproductive lives. To assess these problems, one section asked women whether (yes or no) each of the following factors would be a significant problem for them in seeking healthcare: permission to go for treatment, money for treatment, distance to a health facility, and a desire to not go alone. All these sections on women’s ability were systematically included in all DHS regardless of the country and without an assessment of the robustness and consistency of the instrument. The construction of the indicators of agency among MWRA is shown in Additional file 1.

In terms of gender-based inequality within communities, we derived community-level variables of inequality from individual-level data, as the DHS does not collect cluster-level data. Similar studies on community effects on contraceptive use have aggregated the values of all members of a cluster or Primary Sampling Unit (PSU) to create a community mean [9, 10, 27]. The PSUs constitute a closer approximation of the actual community. The state of discrimination against women was assessed based on the cluster averages for acceptance of domestic violence, marriage before the age of 18, female genital mutilation, unpaid work and high fertility expectations. On the other hand, access to opportunities for women was evaluated based on the cluster averages for house/land ownership, secondary education, exposure to family planning messages, and contact with family planning workers. An operational description of the community-level variables is provided in Additional file 2.

### Control variables

We accounted for the socioeconomic factors in each cluster by adjusting for women’s age and education as well as their household wealth and place.

### Data analyses

The weighting, stratification and clustering factors defined by the DHS were used in analyses, except in component analysis and Cronbach’s alpha testing. Descriptive statistical analyses of mDFPS, empowerment indicators, and socioeconomic factors were performed. All analyses were performed with Stata version 14 [39].

First, we applied principal component analysis and Cronbach’s alpha test to explore the components and assess the consistency of married women’s agency in the Burkina Faso context. We then explored community-level indicators of gender equality by assuming that individuals in the same community were likely to be similar than individuals in different communities [40]. Community averages were dichotomized into “high” and “low” groups based on national medians for descriptive purpose (Table 2); they were used as continuous variable in logistic regression (Table 3). Then, the chi-square test was used to assess bivariate relationships. In addition, the variance inflation factor (VIF) was checked for multicollinearity using a threshold of less than five (< 5).

The hierarchical structure of the DHS data requires the consideration of clustering within communities. The percentage of variance related to clustering is represented by intraclass correlation (ICC), which derived from the null model in which only cluster variable was included as random-effect term. We accounted for unobserved community-level variations using multilevel analysis [40].

The regression strategy involved the fitting of three models. The first (Model 1) included the components of agency in marital relationships. Model 2 included community-level indicators of gender equality as continuous. Model 3 controlled for socioeconomic factors. Robust standard errors were used.

### Ethical considerations

The proposed analysis was exempted from review as described in the enforcement rules issued by the Institutional Review Board of National Yang-Ming University.

## Results

### Sample characteristics

Overall, less than one-third (30.7%) of all 4714 MWRA who had family planning needs used modern methods. Meanwhile, two-thirds (66.9%) used no contraception at all, and 2.4% relied on traditional methods.

### Dimensionality of women’s agency in marital relationships

In the exploration of the dimensionality of agency among married women, three components were retained. These three components matched the three sections of the DHS on participation in household decision-making, problems accessing healthcare and attitudes toward domestic violence; the eigenvalues were 1.7, 2.2, and 3.1, respectively. The evaluation of the internal consistency of each dimension yielded Cronbach’s alpha coefficients of 0.65, 0.83 and 0.70 for participation in household decision-making, problems accessing healthcare and attitudes toward domestic violence, respectively (Table 1). Following this analysis, we grouped each dimension into higher and lower levels of agency. For instance, women who solely or jointly participated in decision-making (regarding family visits, their own healthcare, or household purchases), had no substantial problems accessing healthcare (permission to go, money needed for treatment, distance to the health facility, or a desire to not go alone) or did not agree with domestic violence at all in any of the listed situations (the wife going out without telling her husband, neglecting the children, arguing with her husband, refusing sex, or burning food) were considered to have higher levels of agency (Table 2).

**Table 1:** Factor loadings of the dimensions of women’s agency in marital relationships before varimax rotation and the Cronbach’s alpha test

Women’s agency	Factors loadings (Dimensionality)			Cronbach’s alpha coefficients (Consistency)
	Attitudes toward domestic violence	Participation in household decision-making	Problems accessing healthcare	
**Participation in household decision-making**				0.65
Family visits	0.1586	0.6136	0.1564	
Own healthcare	0.1500	0.5701	0.2019	
Household purchases	0.0980	0.3495	0.2463	
**Problems accessing healthcare**				0.70
Needs permission to go	0.0799	−0.2277	0.4930	
Needs money for treatment	0.0667	−0.0601	0.3568	
Is prohibited by the distance to health facility	0.0973	−0.2318	0.4412	
Is not willing to go alone	0.0913	−0.1962	0.4984	
**Attitudes toward domestic violence**				0.83
If the wife goes out without telling her husband	0.4440	−0.0881	−0.0951	
If the wife neglects the children	0.4536	−0.0946	−0.1087	
If the wife argues with her husband	0.4552	−0.0581	−0.1306	
If the wife refuses sex	0.4087	−0.0345	−0.1354	
If the wife burns food	0.3701	−0.0859	−0.0664	
**Eigen values**	3.1	1.7	2.2	
**Variance explained (%)**	25.7	18.5	14.0

**Table 2:** Demand for family planning satisfied with modern methods (**mDFPS**) in relation to marital relationships, gender inequality in communities, and socioeconomic characteristics

Variables	N	%	mDFPS (%)	***P***-value
**Contraceptive prevalence**				
Modern methods			30.7	
Traditional methods			2.4	
**Women’s agency**	N (4, 714)			
Participation in household decision-making				0.0001
No: No participation (0)	1, 903	39.1	26.5	
Yes: Maybe (1–3)	2, 811	60.9	33.5	
Problems accessing healthcare				0.0001
Yes: Maybe (0–3)	3, 639	77.7	27.8	
No: No problems (4)	1, 075	22.3	41.0	
Attitudes toward domestic violence				0.0001
Agree: Maybe agree (0–4)	2, 040	43.9	26.0	
Opposed: Do not agree (5)	2, 674	56.1	34.4	
**Community-level of gender equality**	N (573)			
*Violence and discrimination against women*				
Acceptance of domestic violence				0.0001
Low	283	49.4	35.7	
High	290	50.6	25.7	
Early marriage				0.0001
Low	285	49.7	38.5	
High	288	50.3	23.8	
Female genital mutilation				0.0025
Low	282	49.2	33.8	
High	291	50.8	27.6	
Unpaid work				0.0001
Low	287	50.1	35.3	
High	286	49.9	26.1	
Fertility expectations				0.0001
Low	287	49.7	38.7	
High	286	50.3	22.0	
*Access to opportunities and resources for women*				
Asset ownership				0.0692
Low	286	49.9	32.6	
High	287	50.1	28.9	
Secondary education				0.0001
Low	284	49.6	21.5	
High	289	50.4	40.0	
Exposure to family planning messages				0.0001
Low	285	49.7	22.8	
High	288	50.3	40.0	
Contact with family planning health worker				0.7080
Low	287	50.1	31.1	
High	286	49.9	30.4	
**Socioeconomic factors**				
**Wealth**				0.0001
Poor	1516	33.9	19.2	
Middle	906	19.0	21.8	
Rich	2292	47.1	42.7	
**Residence**				0.0001
Urban	1522	27.3	48.2	
Rural	3192	72.7	24.2	
**Women’s age**				0.0471
15–24	1162	25.0	27.5	
25–39	1900	40.7	32.0	
40–49	1652	34.3	31.6	
**Women’s education level**				0.0001
No education	3596	76.7	25.3	
Primary	674	13.7	40.5	
Secondary & Higher	444	9.6	60.4	

### Use of modern contraceptives in relation to women’s agency, community-level gender equality, and socioeconomic factors

Overall, 61% of MWRA participated in some family decisions, and 22% of them had no problems accessing healthcare, while 56% did not agree with domestic violence at all. All three dimensions were positively associated with mDFPS. With respect to the community-level indicators of gender equality, there were lower proportions of mDFPS in communities with high levels of acceptance of domestic violence, early marriage, female genital mutilation, unpaid work, fertility preferences, and asset ownership. In contrast, a higher proportion of mDFPS was reported in communities with a high level of secondary education and exposure to family planning messages. For socioeconomic factors, there was a higher proportion of mDFPS reported among women living in wealthier households, women living in urban areas, older women, and more educated women (Table 2).

### Multilevel analysis of predictors of mDFPS

In our regression strategy, we sequentially added components of women’s agency in marital relationships (Model 1), community-level indicators of gender equality regarding discrimination and access to opportunities for women (Model 2), and socioeconomic factors (Model 3). The results are displayed in Table 3.

**Table 3:** Multilevel logistic modeling of women’s empowerment and demand for family planning satisfied using modern methods adjusting for socioeconomic characteristics

Regressions	Model 1		Model 2		Model 3	
Women’s agency	aOR	95% CI	aOR	95% CI	aOR	95% CI
Participation in household decision-making (No)						
Maybe participation	1.30**	[1.11–1.53]	1.20*	[1.03–1.41]	1.09	[0.93–1.28]
Problems accessing healthcare (Maybe)						
No problems	1.62***	[1.36–1.93]	1.45***	[1.22–1.73]	1.27**	[1.06–1.51]
Attitudes toward domestic violence (Agree)						
Opposed	1.36***	[1.16–1.60]	1.23*	[1.04–1.46]	1.13	[0.95–1.35]
***Community-level of gender equality***						
*Violence and discrimination against women*			1.0	[0.63–1.59]	0.91	[0.58–1.44]
Acceptance of domestic violence						
Early marriage			0.91	[0.42–1.96]	0.99	[0.45–2.16]
Female genital mutilation			2.59***	[1.60–4.18]	2.46***	[1.52–3.99]
Unpaid work			0.84	[0.61–1.16]	0.85	[0.61–1.19]
Fertility expectations			0.70***	[0.60–0.82]	0.75***	[0.64–0.87]
*Women’s rights and opportunities*						
Asset ownership			1.67*	[1.10–2.53]	1.72*	[1.13–2.61]
Secondary education			4.91***	[2.08–11.55]	1.98	[0.76–5.20]
Exposure to family planning messages			2.95***	[1.83–4.74]	2.68***	[1.64–4.36]
Contact with family planning health workers			1.40	[0.75–2.63]	1.43	[0.77–2.66]
**Socioeconomic factors**						
**Wealth (Poor)**						
Middle					1.09	[0.87–1.35]
Rich					1.68***	[1.35–2.08]
**Residence (Rural)**						
Urban					0.87	[0.65–1.16]
**Women’s age (15–24)**						
25–39					1.25*	[1.03–1.52]
40–49					1.38**	[1.11–1.71]
**Women’s education level (No education)**						
Primary					1.26*	[1.17–1.85]
Secondary & Higher					1.38**	[1.11–1.72]
**Model statistics**						
Log likelihood		− 2783.11		− 2682.2		− 2659.6
Chi-square		60.4		276.9		335.6
**Comparison to previous model**						
Chi-square				201.7***		110.9***
Degrees of freedom				9		7
**Random variance**						
ICC Null = 0.20 95% CI [0.16–0.24]	0.17	[0.14–0.22]	0.08	[0.06–0.12]	0.07	[0.05–0.12]
Variance between clusters Null: 0.82 95% CI [0.64–1.05]	0.70	[0.54–0.91]	0.29	[0.19–0.44]	0.28	[0.19–0.43]
PVC (%)	15		65		66	

*aOR* Adjusted Odds Ratios, *CI* Confidence Interval, Intra-class correlation (*ICC*) measures the degrees of clustering with random intercepts. The correlation of the 2-level multilevel logistic regressions is calculated by σμ2/ [σμ2 + π2/3], where σμ2 denotes community- level variance; *PVC* Proportional Variance Change; * *p* < 0.05; ** *p* < 0.01; *** *p* < 0.001

Model 1 showed significant positive associations of all three components of agency with mDFPS. The odds of mDFPS increased by 30, 62 and 36% for women who participated in some family decisions, faced no problems accessing healthcare, and were opposed any type of domestic violence, respectively. In Model 2, the addition of community-level indicators decreased the estimates in Model 1; however, the significance and directionality remained. Women in communities with higher prevalence of female genital mutilation (OR: 2.59; 95% CI [1.60–4.18]), were significantly more likely to report mDFPS. In contrast, living in communities with higher fertility expectations (OR: 0.70; 95% CI [0.60–0.82]) lowered the odds of reporting mDFPS. In parallel, residing in a community with higher average access to secondary education (OR: 4.91; 95% CI [2.08–11.55]) and exposure to family planning messages (OR: 2.95; 95% CI [1.83–4.74]) was positively associated with mDFPS. Finally, when socioeconomic factors were added in Model 3, the effect size and the statistical significance of agency components were greatly reduced, but the directionality remained the same. Although opposition to domestic violence and participation in family decisions lost their significance, they were still marginally and positively associated with mDFPS. In contrast, previous estimates of community-level indicators remained essentially unchanged in their significance and directionality, except for secondary education. Furthermore, household wealth, as well as women’s age and education, were positively associated with mDFPS.

The comparison of the three models showed substantial variations in community-level indicators compared to those of the null model (σ2 = 0.82, 95% CI 0.64 to 1.05). For instance, the log likelihood test found that model 2 had a better fit than model 1 [χ2 (9) = 201.7; *p* < 0.001] and that model 3 had a better fit than model 2: [χ2 (7) = 110.9; p < 0.001]. Furthermore, the modeling process showed that the proportions of variance change (PVCs) from the null model were 15, 65 and 66% for models 1, 2, and 3, respectively.

## Discussion

To our knowledge, this is the first study to explore the impact of women’s empowerment on mDFPS, both within marital relationships and in communities in Burkina Faso. As expected, the proportion of mDFPS was suboptimal among MWRA; however, in addition to conventional approaches such as improving accessibility to health services, progress can be made by empowering women and promoting gender equality. Meanwhile, in communities, promoting women’s access to productive means (assets and family planning information) rather than emphasizing reproductive roles (high fertility expectations) may also increase the mDFPS. One finding that was incompatible with the general description above was that living in communities with a higher prevalence of female genital mutilation was associated with increased odds of mDFPS.

### Suboptimal demand for family planning satisfied with modern methods

Less than one-third of MWRA who had family planning needs were able to use modern methods. In Burkina Faso, Choi et al. [15] found 40% of mDFPS, but the pace of the growth was three times slower than that required to reach 75% by 2030.

### Substantial effect of women’s agency on mDFPS

Similar to previous research, we found that participation in household decision-making, a lack of problems accessing healthcare, and negative attitudes toward domestic violence constituted separate components of women’s agency [33, 41]. Moreover, the variables for each component were fairly consistent within our study population. In addition, three-fifths of the MWRA either were able to participate in household decisions or held negative views on domestic violence, while only one-fifth had no problems accessing healthcare. According to Wayack-Pambe, few married women in Burkina Faso are empowered; only 59% of them participate in decision-making, while 61% live under psychological pressure [42]. Furthermore, we found that the odds of mDFPS increased with the presence of each component of women’s agency, especially the freedom to access healthcare. The associations between women’s agency and contraception have been studied fairly extensively and seem to be mostly positive [35, 36]. However, no study has systematically accounted for the dimensionalities and consistency of indicators of agency as related to mDFPS. Considering these parameters in our study not only strengthened the results but also revealed the struggle that MWRA have undergone to avoid unintended pregnancies. In addition, our study may have identified freedom in accessing healthcare as a contextually salient component in the relationship of women’s agency with mDFPS. However, previous conceptualizations of empowerment have considered women’s freedom of movement to be unlimited in the sub-Saharan African context [43, 44]. In fact, heath-seeking behavior is not trivial for married women, as it requires financial independence and more equal spousal power dynamics. In Burkina Faso, previous research found that married women with home ownership or bargaining power were more likely to meet their healthcare needs that were less obvious to husbands, including contraception [19, 45]. It should be noted that these components of agency are also influenced by levels of inequality in communities [42].

Adding community-level of gender equality reduced the influence of women’s agency on mDFPS especially participation in household decision-making and attitudes toward domestic violence. Previous research suggested that in communities that emphasized on traditional gender norms and relationships, perceived benefit gained from using family planning diminish [46]. In fact, it is possible that women who participate in household decisions and have equitable gender norms may more carefully consider, beyond the health risks, the social costs of losing their independence due to modern contraceptive adoption. In contrast, the lack of significant reduction on the effect of women’s access to healthcare may indicate that there is a weaker control of community gender norms and relationships on women’s health seeking behavior [47]. Conversely, Yaya et al. found in sub-Saharan Africa that women who have high decision making power and medium acceptance of wife beating still have increased odds of ever use of contraception, after controlling for community and country levels of socioeconomic factors [26]. However, the authors focused on past experience of contraception regardless of the type of methods. This approach may have overlooked the effects of contraceptive past experience on the process of empowerment. In addition, seeking modern and traditional methods of contraception may involve different levels of empowerment. Furthermore, our findings emphasized on the need to consider the effects of gender equality in communities rather than that of crude socioeconomic factors in relation with modern contraceptive use.

### Beneficial effects of gender equality in communities on mDFPS

Consistent with socioecological theories, community-level gender inequality also shaped mDFPS. Previous studies found that discrimination against women negatively impacted modern contraceptive use, while greater opportunity for women had the opposite effect [10, 27, 28]. We found substantial variation in mDFPS across communities. Women in communities with higher fertility expectations were significantly less likely to address their family planning needs with modern methods. This finding points to African pronatalism as the most common and salient obstacle to modern contraception, as it prioritizes women’s reproductive roles and denies women the choice to limit their fertility [9, 48]. Through social conformity, women may adhere to the prevailing fertility expectations and refrain from using modern contraceptives.

Furthermore, women may conform to the prevailing fertility norms to gain social approval and avoid violence. Consistent with previous research, we did not find significant relationship between violence justification at the community level and modern contraceptive use [10]. Despite an overall negative association between acceptance of domestic violence and modern contraceptive use in African communities, within countries disparities may still exist [9]. In fact, the impact of both acceptance and experience of violence on women’s contraceptive behavior is complex to capture due to stigma which may lead to under-reporting. Further work is needed to develop tools that can capture specific and reliable elements of violence in communities.

Unexpectedly, a higher practice of female genital mutilation in the community was positively associated with the use of modern contraception. We ruled out the possibility of the ecological fallacy (Additional file 3) by controlling for individual-level genital mutilation status. As female genital mutilation is seen as an indicator of the violation of women’s rights, we expected to find a negative association with modern contraception [49]. Although the practice of female genital mutilation is discriminatory against women, women may have undergone the procedure as an act of conformity, and there is even a sense of pride in the practice in many African societies, including Burkina Faso [50, 51]. Therefore, female genital mutilation may not necessarily deter a household from using modern contraceptives. On the other hand, it is possible that in communities where the practice of female genital mutilation is widespread, the complications of pregnancies and childbirths are better understood and may have encouraged women to adopt more effective contraceptive measures [52]. Further research is required to examine the impact of other community-level influences that may mediate the association between female genital mutilation and modern contraceptive use.

Unsurprisingly, our findings on the association between community-level access to rights and resources for women and mDFPS were consistent with the current literature [9, 10, 27]. In particular, we found that greater access to assets and exposure to family planning messages in the community was also associated with higher odds of mDFPS. Our results indicate independent effects of community-level rights and opportunities for women as well as violence and discrimination against women on mDFPS. Therefore, the expansion of mDFPS should take into account both community factors, such as political changes, and cultural barriers regarding gender equality [53]. In addition, women’s access to rights and resources relative to those of men should also be considered. In fact, an increased male-to-female ratio may reflect higher gender inequality and privileging of the reproductive over the productive roles of women [27].

### Limitations and strengths

This study used a socioecological framework, a systematic analytical strategy, and rigorous statistical methods to assess mDFPS and its link to women’s empowerment. Nonetheless, we acknowledge several limitations. Due to a lack of data, we were unable to control for the community-level of access to family planning, which remains unexplained. Additionally, most of the empowerment indicators in the DHS questionnaire were developed in South Asia [54]. Therefore, the empowerment indicators may not adequately reflect the Burkina Faso context. Future research may examine ethnographic evidence and use more appropriate variables. However, our results are consistent with those of previous studies in a similar context. Moreover, as a cross-sectional study, this analysis does not support any inferences about causality in the relation between women’s empowerment and modern contraceptive use. A longitudinal study may enhance our understanding of the dynamic interactions between the dimensions of empowerment and socioeconomic factors. Last, community-derived gender inequality indicators are based on physical boundaries, which may not fully represent the concept of community in terms of sociocultural entity [55].

Despite the limitations, this study also has strengths. First and foremost, the nationwide survey with representative sampling allowed multilevel analyses, which provided a more comprehensive outline for understanding the influence of gender inequality on women’s reproductive behavior, especially in resource-constrained countries. This study conceptualized power dynamics at multiple levels and further identified multiple and relevant components of women’s agency in the Burkina Faso context. Such results could be useful in the future conceptualization, measurement and interpretation of empowerment. As the study population was limited to those with some interest in family planning per the selection criteria, we might have neglected the need for family planning in the non-selected sub-population. We therefore compared woman’s agency, empowerment and socioeconomic indicators between the selected and non-selected groups (Additional file 4). We found that non-selected married women of reproductive age reported significantly lower levels of participation in household decision-making, freedom in accessing healthcare and opposition of domestic violence. Moreover, we also found significantly higher proportion of non-selected women living in communities with high levels of acceptance of domestic violence, early marriage, female genital mutilation, unpaid work and fertility expectations. In contrast, non-selected women more frequently lived in communities with low levels of secondary education, and exposure to family planning messages. Women in the non-selected group were also significantly poorer, younger, less educated and more concentrated in rural areas. It is possible that these women had not perceived family planning as desirable or possible, and therefore the need for empowering women and promoting gender equality may be greater than the results of this study suggest. Last, this study provided insight into the need to adopt a multilevel and comprehensive approach to addressing socioeconomic development, gender inequality and sexual and reproductive rights.

## Conclusions

Gender inequality and power imbalances in marital relationships limit women’s ability to participate in strategic life choices, including the decision to use modern methods when family planning is needed. The findings of this original study highlight a range of indicators or factors at multiple levels that should be considered, not only for family planning interventions but also for gender, population and development policies. In fact, to accelerate progress toward universal access to family planning, interventions need to maximize the impact on women’s empowerment. Therefore, an integrated approach is necessary to simultaneously target the prevailing fertility and gender norms operating at the community level and promote shared household decisions.

## Supplementary Information

**Additional file 1.** Indicators of women’s agency in marital relationships derived from the DHS responses.

**Additional file 2.** Operational description of the community-level variables included in the analyses.

**Additional file 3.** Model 3 regression including individual and community levels of female genital mutilation.

**Additional file 4.** Comparative table of selected women (“with current need for family planning”) and non-selected women (“without current need for family planning”) on variables related to agency, community gender equality and socioeconomic characteristics.

## Data Availability

The data supporting the findings of this research are publicly available from the DHS Program in https://dhsprogram.com/

## Abbreviations

SDGs: Sustainable Development Goals
MWRA: Married Women of Reproductive Age
mDFPS: Demand for Family Planning Satisfied with modern methods
DHS: Demographic and Health Survey
PSU: Primary Sampling Unit
aOR: adjusted Odd Ratio
PCA: Principal Component Analysis
ICC: IntraClass Correlation
PVC: Proportion of Variance Change
