# Supplementary material for: Empowerment and use of modern contraceptive methods among married women in Burkina Faso: a multilevel analysis
Source: BMC Public Health. 2021 Aug 3;21:1498. doi: 10.1186/s12889-021-11541-x (PMC8336087; doi:10.1186/s12889-021-11541-x)
Supplement: Supplementary file 1 — Additional file 1. Indicators of women’s agency in marital relationships derived from the DHS responses. [file 12889_2021_11541_MOESM1_ESM.docx]

**Appendix file 1.** Indicators of women’s agency in marital relationships derived from the DHS responses

| **Indicator** | **DHS question** | **Original categorization from the DHS** |
| --- | --- | --- |
| **Participation in household decision-making** | Who usually has the final say in the family in the following decisions? (3 responses **^b^**) | 1 = respondent alone  2 = jointly  4 = partner alone  5 = someone else  6 = other |
| **Problems accessing healthcare** | When you are sick and want to get medical advice or treatment, is each of the following a big problem or not? (4 responses **^c^**) | 1= big problem  2 = not a big problem |
| **Attitudes toward domestic violence** | Is a husband justified in hitting/beating his wife in the following situations? (5 responses **^a^**) | 1 = yes  0 = no  8 = don’t know |
| a. If a wife goes out without telling him, neglects the children, argues with him, refuses sex, or burns food.  b. Decisions on family visits, one’s own healthcare, or household purchases  c. Permission to go, money needed for treatment, distance to the health facility, or desire to not go alone | | |
