# Supplementary material for: Empowerment and use of modern contraceptive methods among married women in Burkina Faso: a multilevel analysis
Source: BMC Public Health. 2021 Aug 3;21:1498. doi: 10.1186/s12889-021-11541-x (PMC8336087; doi:10.1186/s12889-021-11541-x)
Supplement: Supplementary file 2 — Additional file 2. Operational description of the community-level variables included in the analyses. [file 12889_2021_11541_MOESM2_ESM.docx]

**Appendix file 2.** Operational description of the community-level variables included in the analyses

| **Community of gender equality** |  |
| --- | --- |
| *Violence and discrimination against women* |  |
| **Acceptance of domestic violence** | Proportion of women aged 15-49 years in the community who agreed with at least one of the following reasons for domestic violence: a wife going out without telling her husband, neglecting the children, arguing with her husband, refusing sex, or burning food. |
| **Early marriage** | Proportion of women aged 15-49 years in the community who married under 18 years of age |
| **Female genital mutilation** | Proportion of women aged 15-49 years in the community who have been mutilated |
| **Unpaid work** | Proportion of women aged 15-49 years who were employed in the last 12 months in the community but were not paid at all |
| **Fertility expectations** | Mean ideal number of children among women aged 15-49 years in the community |
| *Access to rights and resources for women* |  |
| **Asset ownership** | Proportion of women aged 15-49 years in the community who owned either a house or land |
| **Secondary education** | Proportion of women aged 15-49 years in the community with at least a secondary education |
| **Exposure to family planning messages** | Proportion of women aged 15-49 years in the community who had been exposed to family planning messages on radio, TV or newspapers |
| **Contact with family planning health worker** | Proportion of women aged 15-49 years in the community who had contact with a family planning worker either at health facility or in community |
