# Supplementary material for: Empowerment and use of modern contraceptive methods among married women in Burkina Faso: a multilevel analysis
Source: BMC Public Health. 2021 Aug 3;21:1498. doi: 10.1186/s12889-021-11541-x (PMC8336087; doi:10.1186/s12889-021-11541-x)
Supplement: Supplementary file 3 — Additional file 3. Model 3 regression including individual and community levels of female genital mutilation. [file 12889_2021_11541_MOESM3_ESM.docx]

**Appendix file 3.** Model 3 regression including individual and community levels of female genital mutilation

| **Regressions** | Model 3 | |
| --- | --- | --- |
| **Women’s agency** | aOR | 95% CI |
| Participation in household decisions (No) |  |  |
| Maybe | 1.09 | [0.93-1.28] |
| Problems accessing healthcare (Maybe) |  |  |
| No | 1.27** | [1.06-1.51] |
| Attitudes toward domestic violence (Agree) |  |  |
| Opposed | 1.13 | [0.95-1.35] |
| **Community-level indicators of gender equality** |  |  |
| *Violence and discrimination against women* |  |  |
| Acceptance of domestic violence | 0.91 | [0.58-1.44] |
| Early marriage | 0.99 | [0.45-2.16] |
| *Female genital mutilation* | *2.49*** | *[1.48-4.20]* |
| Unpaid work | 0.85 | [0.61-1.19] |
| Fertility expectation | 0.75*** | [0.64-0.87] |
| *Women’s rights and opportunities* |  |  |
| Asset ownership | 1.72* | [1.13-2.62] |
| Secondary education | 1.99 | [0.76-5.18] |
| Exposure to family planning messages | 2.68*** | [1.64-4.36] |
| Contact with family planning health workers | 1.43 | [0.77-2.66] |
| **Socioeconomic factors** |  |  |
| **Wealth (Poor)** |  |  |
| Middle | 1.09 | [0.87-1.35] |
| Rich | 1.68*** | [1.35-2.08] |
| **Residence (Rural)** |  |  |
| Urban | 0.87 | [0.65-1.16] |
| **Women’s age (15-24)** |  |  |
| 25-39 | 1.26* | [1.04-1.53] |
| 40-49 | 1.38** | [1.12-1.71] |
| **Women’s education level (No education)** |  |  |
| Primary | 1.47** | [1.17-1.84] |
| Secondary & Higher | 2.33*** | [1.73-3.16] |
| ***Genital mutilation (Ref: Yes)*** |  |  |
| *No* | *1.01* | *[0.81-1.26]* |
| **Model statistics** |  |  |
| Log likelihood |  | -2640.0 |
| Chi-square |  | 345.8 |
| **Random variance** |  |  |
| ICC Null= 0.20 95% CI [0.16-0.24] | 0.08 | [0.05-0.12] |
| Variance between clusters  Null: 0.82 95% CI [0.64-1.05] | 0.28 | [0.19-0.43] |
| **Note: aOR= Adjusted Odds Ratios, CI = Confidence; Interval, Intra-class correlation (ICC) measures the degrees of clustering with random intercepts. The correlation of the 2-level multilevel logistic regressions is calculated by σμ2/ [σμ2 + π2/3], where σμ2 denotes community- level variance; * p<0.05; ** p<0.01; *** p<0.001** | | |
