# Supplementary material for: Empowerment and use of modern contraceptive methods among married women in Burkina Faso: a multilevel analysis
Source: BMC Public Health. 2021 Aug 3;21:1498. doi: 10.1186/s12889-021-11541-x (PMC8336087; doi:10.1186/s12889-021-11541-x)
Supplement: Supplementary file 4 — Additional file 4. Comparative table of selected women (“with current need for family planning”) and non-selected women (“without current need for family planning”) on variables related to agency, community gender equality and socioeconomic characteristics. [file 12889_2021_11541_MOESM4_ESM.docx]

**Appendix file 4**. Comparative table of selected women ("with current need for family planning") and non-selected women ("without current need for family planning") on variables related to agency, community gender equality and socioeconomic characteristics

| **Variables** | **N** | **%** | **Non-selected women (%)** | **Selected women (%)** | **P-value** |
| --- | --- | --- | --- | --- | --- |
| **No need for family planning** | 6321 | 57.5 |  |  |  |
| **Need for family planning** | 4714 | 42.5 |  |  |  |
| **Women’s agency** |  |  |  |  |  |
| Participation in household decision-making |  |  |  |  | 0.0020 |
| No: No participation (0) | 4680 | 41.1 | 42.7 | 39.1 |  |
| Yes: Maybe (1-3) | 6355 | 58.9 | 57.3 | 60.9 |  |
| Problems accessing healthcare |  |  |  |  | 0.0002 |
| Yes: Maybe (0-3) | 8732 | 79.7 | 81.2 | 77.7 |  |
| No: No problem (4) | 2303 | 20.3 | 18.8 | 22.3 |  |
| Attitudes toward domestic violence |  |  |  |  | 0.0094 |
| Agree: Maybe agree (0-4) | 4979 | 45.7 | 47.0 | 43.9 |  |
| Opposed: Do not agree (5) | 6056 | 54.3 | 53.0 | 56.1 |  |
| **Community-level of gender equality** | 573 clusters |  |  |  |  |
| *Violence and discrimination against women* |  |  |  |  |  |
| Acceptance of domestic violence |  |  |  |  | 0.0001 |
| Low | 283 | 49.4 | 43.0 | 50.2 |  |
| High | 290 | 50.6 | 57.0 | 49.8 |  |
| Early marriage |  |  |  |  | 0.0001 |
| Low | 285 | 49.7 | 36.3 | 47.5 |  |
| High | 288 | 50.3 | 63.7 | 52.5 |  |
| Female genital mutilation |  |  |  |  | 0.0001 |
| Low | 282 | 49.2 | 43.0 | 50.8 |  |
| High | 291 | 50.8 | 57.0 | 49.2 |  |
| Unpaid work |  |  |  |  | 0.0754 |
| Low | 287 | 50.1 | 48.4 | 50.8 |  |
| High | 286 | 49.9 | 51.6 | 49.2 |  |
| Fertility expectations |  |  |  |  | 0.0001 |
| Low | 287 | 49.7 | 37.6 | 52.4 |  |
| High | 286 | 50.3 | 62.4 | 47.6 |  |
| *Access to opportunities and resources for women* |  |  |  |  |  |
| Asset ownership |  |  |  |  | 0.1762 |
| Low | 286 | 49.9 | 48.3 | 50.3 |  |
| High | 287 | 50.1 | 51.7 | 49.7 |  |
| Secondary education |  |  |  |  | 0.0001 |
| Low | 284 | 49.6 | 65.6 | 50.0 |  |
| High | 289 | 50.4 | 34.5 | 50.0 |  |
| Exposure to family planning messages |  |  |  |  | 0.0054 |
| Low | 285 | 49.7 | 57.8 | 53.8 |  |
| High | 288 | 50.3 | 42.2 | 46.2 |  |
| Contact with family planning health worker |  |  |  |  | 0.4653 |
| Low | 287 | 50.1 | 50.0 | 51.1 |  |
| High | 286 | 49.9 | 50.0 | 48.9 |  |
| **Socioeconomic factors** |  |  |  |  |  |
| **Wealth** | 287 | 50.1 |  |  | 0.0001 |
| Poor | 286 | 49.9 | 43.1 | 33.9 |  |
| Middle | 2318 | 20.8 | 22.2 | 19.0 |  |
| Rich | 4593 | 40.0 | 34.7 | 47.1 |  |
| **Residence** |  |  |  |  | 0.0001 |
| Urban | 2805 | 20.9 | 16.1 | 27.3 |  |
| Rural | 8230 | 79.1 | 83.9 | 72.7 |  |
| **Women’s age** |  |  |  |  | 0.0001 |
| 15-24 | 3418 | 31.7 | 36.8 | 25.0 |  |
| 25-39 | 4748 | 42.7 | 44.1 | 40.7 |  |
| 40-49 | 2869 | 25.6 | 19.1 | 34.3 |  |
| **Women’s education level** |  |  |  |  | 0.0001 |
| No education | 8892 | 81.3 | 84.8 | 76.7 |  |
| Primary | 1343 | 11.5 | 9.9 | 13.7 |  |
| Secondary & Higher | 800 | 7.2 | 5.3 | 9.6 |  |
